# Comparative Evaluation of the Anti-bacterial Efficacy of Herbal Medicaments and Synthetic Medicaments Against Enterococcus faecalis using Real-time Polymerase Chain Reaction

**DOI:** 10.7759/cureus.5228

**Published:** 2019-07-24

**Authors:** Rajeswari Kalaiselvam, Karthick Soundararajan, Mathan Rajan R, Kandaswamy Deivanayagam, Chakravarthy Arumugam, Arathi Ganesh

**Affiliations:** 1 Conservative Dentistry and Endodontics, Faculty of Dental Sciences, Sri Ramachandra University, Chennai, IND

**Keywords:** herbal medicaments, synthetic medicaments, enterococcus faecalis, real-time polymerase chain reaction

## Abstract

Periradicular bacterial infections are the proven cause of the failure of endodontic treatment. When the pulp canal gets infected or becomes necrotic, bacterial growth takes place in the form of biofilms and aggregates. During the endodontic procedure, bacterial colonies are disrupted, and the microbial load is reduced by biomechanical preparation, cleaning with anti-microbial solutions, and placing anti-bacterial medicaments in the root canal. These anti-microbial substances are synthetic, having a cytotoxicity effect. In recent times, herbal medicines are an alternative medicine being used increasingly as an intra-canal medicament to fight or prevent common endodontic infections.

Aim

The objective of this study is to compare the anti-bacterial efficacy of herbal medicaments and synthetic medicaments against Enterococcus faecalis (E. faecalis).

Materials and methods

This was an in-vitro study in which a total of 60 teeth specimens (n=60) were inoculated with E. faecalis for 21 days. Specimens were divided into six groups (Group 1: Piper nigrum (Piperaceae); Group 2: Piper longum (Piperaceae); Group 3: Zingiber officinale Roscoe (Zingiberaceae); Group 4: Calcium hydroxide; Group 5: 2% chlorhexidine gel (CHX); and Group 6: saline (negative control). The intra-canal medicaments were packed inside the tooth. After five days, the remaining microbial load was determined by using real-time PCR.

Results

The threshold cycle (Ct) values of Piper nigrum, Piper longum, dried ginger extract, 2% CHX, calcium hydroxide, and saline were found to be 21.36, 20.55, 22.14, 22.51, 23.62, and 17.81, respectively. The obtained mean bacterial load of these products was 8.64, 12.52, 7.23, 0.82, 0.14, and 149.49, respectively.

Conclusion

Two percent CHX showed high antibacterial activity against E. faecalis followed by calcium hydroxide, Zingiber officinale Roscoe, Piper nigrum, Piper longum, and saline.

## Introduction

Periradicular infections often contribute to the failure of endodontic treatment. [1] Enterococci is the predominant microorganism in the root canal, which is gram-positive and facultative anaerobe [2] and majorly responsible for perioapical lesions. About 77% of persistent endodontic infections are asymptomatic [2-6] and possess certain virulent factors like proteolytic enzymes, cytolysin, aggregation substance, pheromones, and lipoteichoic acid. They have a tendency for bonding to the root canal walls and forming a biofilm [7]. They survive a wide variety of growth conditions, including a temperature range of 10°C to 45°C, and they also live in hypotonic, hypertonic, acidic, or alkaline environments [8] Various studies have shown that Enterococci are resistant to various intracanal treatment procedures [9]. This is attributed to their ability to penetrate dentinal tubules, withstand high pH values, possess virulence factors, and form biofilm [10]. Canal preparation does not completely eliminate the bacteria, but the application of canal medications can eliminate the persistent organisms following biomechanical preparation.

Mechanical preparation of the canal leads to disruption in the microbial configuration, whereas anti-microbial rinsing of the canal results in a reduction in the microbial load [11]. Herbal derivatives are a good alternative medicament having anti-microbial potent due to the amalgamation of alkaloids present in these mixtures [12]. This study highlights the efficacy of a herbal product as an intracanal medicament to overcome the cytotoxicity of polypotent stem cells. This study is done to evaluate the antibacterial efficacy of dried fruits of Piper nigrum (Piperaceae), Piper longum (Piperaceae), and rhizome of Zingiber officinale Roscoe (Zingiberaceae) extracts as an intracanal medicament against E. faecalis by using real-time polymerase chain reaction (PCR) tests [13].

## Materials and methods

Preparation of the blocks

Sixty freshly extracted non-carious, mandibular premolar teeth were selected for the study. Decoronation of the teeth was done with a rotary diamond disk and the middle third of the root was taken for the study purpose. The dentin blocks were standardized to its internal diameter to GG drill no.3 (Mani Inc, Tachigiken, Japan). The blocks were treated with 3% sodium hypochlorite for 10 minutes followed by 17% ethylene diamine tetraacetic acid for five minutes to remove the smear layer. The blocks were rinsed with 10 ml distilled water to eliminate the residual irrigant solution and subjected to autoclave sterilization at 121°C.

Contamination of the dentin blocks

The antibacterial activity of the test medicament was tested with a pure strain of Enterococcus faecalis using American Type Culture Collection and laminar flow method. Overnight, an incubated fresh culture of E. faecalis was grown in a brain heart infusion broth, incubated for 24 hours at 37°C. One ml of inoculums was transferred to individual sterile microcentrifuge tubes containing 1 ml of the respective broths and dentin block. After 48 hours, fresh broth containing microorganism was transferred to the tube containing dentin blocks.

Anti-microbial assessment

After the incubation period, 5 ml of sterile saline was used as an irrigant to eliminate the broth medium. The dentin blocks were grouped as follows (n=10):

Group 1: Piper nigrum (Piperaceae) extract with methylcellulose

Group 2: Piper longum (Piperaceae) extract with methylcellulose

Group 3: Zingiberofficinale Roscoe (Zingiberaceae) extract with methylcellulose

Group 4: Calcium hydroxide

Group 5: 2% chlorhexidine gel

Group 6: Untreated positive control

After treating with medication, all the blocks were covered with a sealant and placed in the aerobic incubator at 37°C. After 24 hours, an antimicrobial assay was carried out with 10 blocks from each group. Using 5 ml sterile saline, the blocks were washed. Dentin debris was harvested at a depth of 400 um by using GG drills no. 5, collected in 1 ml of sterile PBS buffer solution, and subjected to aerobic incubation at 37C for 24 hours. Following this procedure, the samples were evaluated by real-time PCR.

## Results

**Figure 1:**
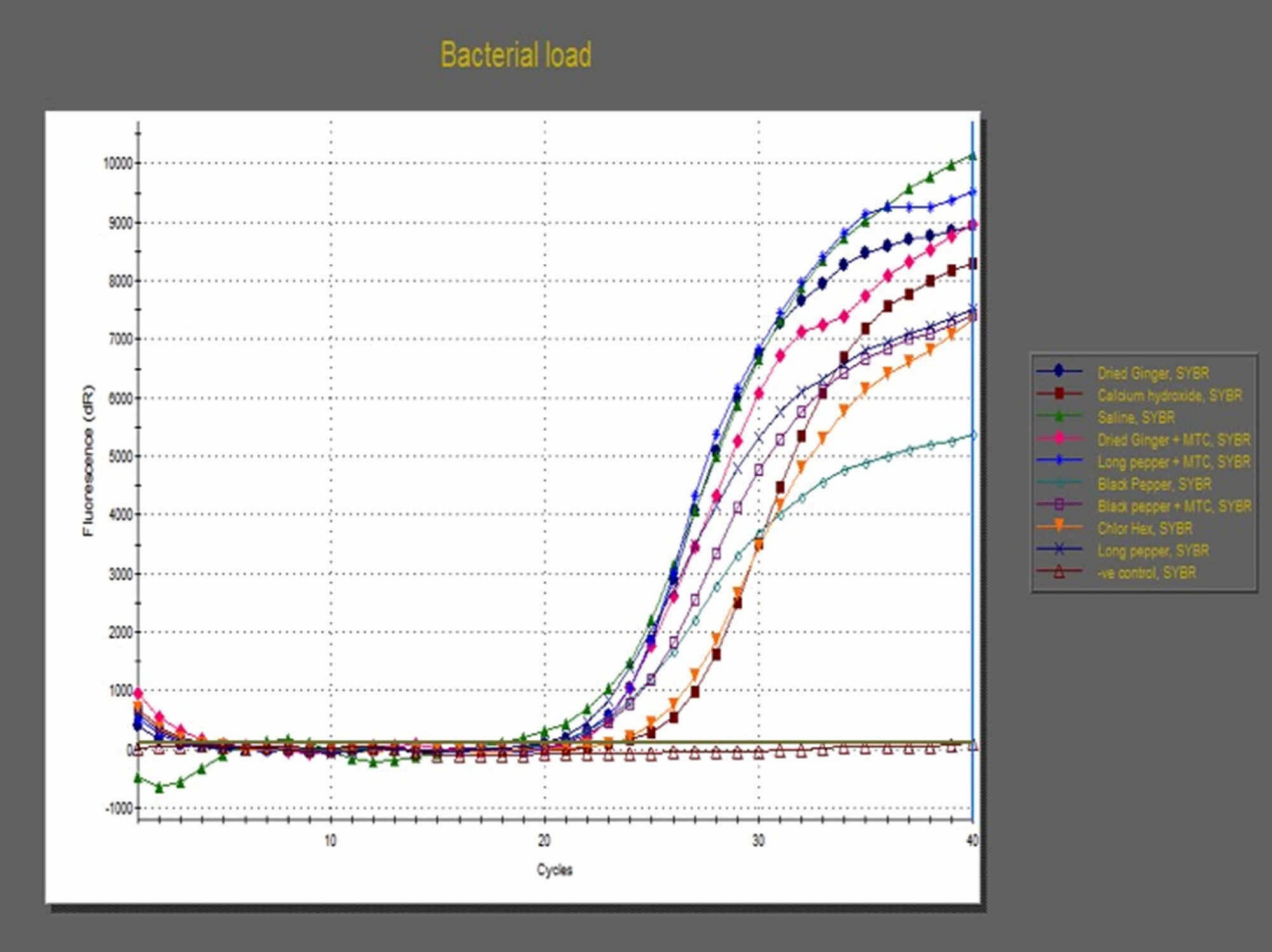
Quantitative real-time polymerase chain reaction (PCR) showing bacterial load

In the results, threshold cycles (Ct) were determined by PCR (Figure 1). The study revealed that 2% CHX has a greater antimicrobial effect, followed by Ca(OH)_2 _(Table 1). It also established that the antibacterial property of Zingiberofficinale Roscoe (Zingiberaceae) extract with methylcellulose was at a satisfactory level. It was calculated that the percentage of bacterial loads was reduced in comparison to saline that is used as a negative control. Table 2 showed the multiple comparison of the mean threshold cycle (Ct) among groups. Two percent chlorhexidine showed the best antibacterial efficacy followed by Ca(OH)_2_ and Zingiberofficinale Roscoe extract with methylcellulose (Table 3). Table 4 shows the multiple comparison of the mean number of copies of bacteria (bacterial load) among groups.

**Table 1:** Comparison of mean threshold cycle (Ct) among the groups *One-way analysis of variance (ANOVA), p<0.05

Group	Mean±SD	F value	p-value
Black Pepper with Methylcellulose	21.369±0.250	149.280	0.000^*^
Long Pepper with Methylcellulose	20.555±0.300		
Dried ginger with Methylcellulose	22.148±0.509		
Calcium hydroxide	22.512±0.330		
Chlorhexidine	23.622±0.913		
Saline	17.816±0.522		

**Table 2:** Multiple comparison of mean threshold cycle (Ct) among groups #Tukey’s honestly significant difference (HSD), p<0.05

Groups	Mean difference	p-value
Black Pepper with methylcellulose* Long Pepper with methylcellulose	0.814	0.012#
Long Pepper with methylcellulose* Dried Ginger with methylcellulose	-1.593	0.000#
Dried Ginger with methylcellulose* Calcium Hydroxide	-0.364	0.627
Calcium Hydroxide* Chlorhexidine	-1.110	0.000#
Chlorhexidine*Saline	5.806	0.000#
Saline*Black Pepper with methylcellulose	-3.553	0.000#
Black Pepper with methyl cellulose*Dried Ginger with methylcellulose	-0.779	0.018#
Black Pepper with methylcellulose*Calcium Hydroxide	-1.143	0.000#
Black Pepper with methylcellulose*Chlorhexidine	-2.253	0.000#
Long Pepper with methylcellulose*Calcium Hydroxide	-1.957	0.000#
Long Pepper with methyl cellulose*Chlorhexidine	-3.067	0.000#
Long Pepper with methylcellulose*Saline	2.739	0.000#
Dried ginger with methylcellulose* Chlorhexidine	-1.474	0.000#
Dried ginger with methylcellulose*Saline	4.332	0.000#
Calcium Hydroxide*Saline	4.696	0.000#

**Table 3:** Comparison of mean number of copies of bacteria (bacterial load) among the groups *One-way analysis of variance (ANOVA), p<0.05

Group	Mean±SD	F value	p-value
Black Pepper with methylcellulose	8.645±0.446	49754.191	0.000
Long Pepper with methylcellulose	12.520±0.498		
Dried ginger with methylcellulose	7.239±0.490		
Calcium Hydroxide	0.827±0.236		
2% Chlorhexidine	0.141±0.113		
Saline	149.49±1.847		

**Table 4:** Multiple comparison of mean number of copies of bacteria (bacterial load) among groups #Tukey’s honestly significant difference (HSD), p<0.05

Group	Mean difference	p-value
Black Pepper with methylcellulose* Long Pepper with methylcellulose	-3.875	0.000#
Long Pepper with methylcellulose*Dried ginger with methylcellulose	5.280	0.000#
Dried ginger with methylcellulose*Calcium Hydroxide	6.412	0.000#
Calcium Hydroxide*Chlorhexidine	0.685	0.450
Chlorhexidine*Saline	-149.35	0.000#
Saline*Black Pepper with methylcellulose	140.85	0.000#
Black Pepper with methylcellulose*Dried ginger with methylcellulose	1.405	0.005#
Black Pepper with methylcellulose*Calcium Hydroxide	7.817	0.000#
Black Pepper with methylcellulose*Chlorhexidine	8.503	0.000#
Long Pepper with methylcellulose*Calcium Hydroxide	11.692	0.000#
Long Pepper with methylcellulose*Chlorhexidine	12.378	0.000#
Long Pepper with methylcellulose*Saline	-136.97	0.000#
Dried ginger with methylcellulose* Chlorhexidine	7.098	0.000#
Dried ginger with methylcellulose*Saline	-142.25	0.000#
Calcium Hydroxide*Saline	-148.66	0.000#

## Discussion

Endodontic treatment success relies on the complete excavation of infective bacteria from the root canal space [14]. The endodontic infection mainly comprises the ecology of different microbial species. Proper cleaning and shaping of the root canal space will eliminate microbial populations [15].

E. faecalis is the most common pathogen associated with both primary and secondary endodontic infections [2-6]. There are various factors involving in the localization of microbes in the host such as oxygen, nutrient supply, bacterial synergism, etc.

E. faecalis form a biofilm, which in turn helps the bacteria to be more resistant towards microbial destruction. Contributing factors to resistance include the impenetrable polysaccharide coating on the biofilm bacteria, and these biofilm bacteria survive without dividing. Various physical factors that favor bacterial growth, which includes pH, ion concentration, nutrient availability, and oxygen supply, vary throughout the biofilm [16].

The main use of an intracanal medicament is to destroy the bacteria that remains even after biomechanics preparation, thereby providing a microbe-free environment. Intracanal medicaments have been used to disinfect root canals. The disinfectants used in the intracanal are camphorated monochlorophenol, formocresol, glutaraldehyde, and halides, as well as other material, including calcium hydroxide (Ca(OH)) and a few antibiotics [17].

The high pH (11-12.5) of Ca(OH) has antimicrobial properties and its dissociation into highly interactive and lethal hydroxyl ions destroys the bacterial cells through protein denaturation and cytoplasmic membrane and DNA disruption. Calcium hydroxide plays a major role in preventing bacterial reinfection by denying the nutrient supply and thus delaying recontamination [18].

The CHX gel has antimicrobial activity for up to 21 days after contamination. The interaction of the positively charged CHX molecules and the negative phosphate ions on the bacterial cell walls leads to the altered osmotic equilibrium of the bacterial cells. This, in turn, increases cell wall diffusion, thereby allowing the CHX molecule to penetrate the bacterial cell.

The purpose of this study is to analyze the antimicrobial property of herbal products and compare it with that of the synthetic antimicrobial agents used as canal medicaments in endodontics. This will lessen the antibiotic resistance as well as provide cost-effective, biocompatibility, and non-toxic benefits.

Piper nigrum (Piperaceae) extract with methylcellulose, Piper longum (Piperaceae) extract with methylcellulose, and Zingiberofficinale Roscoe (Zingiberaceae) extract with methylcellulose are the herbal products used for the study. The study results revealed the better antibacterial efficacy of 2% CHX with Ca(OH)_2_ and dried ginger when combined with methylcellulose as a base. Two percent CHX is a bactericidal medicament having the ability to diffuse through the dentinal tubules. Gingerol is the major active ingredient of ginger and is responsible for the antimicrobial properties.

The advantage of real-time quantitative PCR lies in discriminating and quantifying live and dead bacteria in the samples. This leads us to consider its application to be of great importance in identifying vulnerable bacteria. Real-time quantitative PCR is very sophisticated and identifies various microbial species and their strains.

## Conclusions

Based on this study, we conclude that 2% chlorhexidine has greater antibacterial efficacy against E. faecalis and Zingiberofficinale Roscoe (Zingiberaceae) extract. Methylcellulose base also possesses similar antibacterial activity against E. faecalis and can be considered for use as a canal medicament in endodontic practice. However in-vivo studies are essential to validate the results.
